# Vitamin D Deficiency Rickets Mimicking Pseudohypoparathyroidism

**DOI:** 10.4274/jcrpe.v2i4.173

**Published:** 2010-11-08

**Authors:** Leyla Akın, Selim Kurtoğlu, Aysel Yıldız, Mustafa Ali Akın, Mustafa Kendirici

**Affiliations:** 1 Department of Pediatrics, Erciyes University Faculty of Medicine, Kayseri, Turkey; +90 352 438 00 76+90 533 240 16 43leylabakin@gmail.comErciyes University, Faculty of Medicine, Department of Pediatric Endocrinology, 38039, Kayseri, Turkey

**Keywords:** Vitamin D deficiency rickets, Pseudohypoparathyroidism, antiepileptic drugs

## Abstract

Vitamin D deficiency rickets (VDDR) is a disorder biochemically characterized by elevated serum alkaline phosphatase (ALP) activity, normal or decreased serum calcium (Ca) and inorganic phosphate concentrations, secondary hyperparathyroidism and decreased serum 25−hydroxyvitamin D (25(OH)D) levels. In stage 1 VDDR, urinary amino acid and phosphate excretion are normal with minimal or no findings of rickets on radiographs. Pseudohypoparathyroidism (PHP) is an inherited disorder characterized by end−organ resistance to parathormone (PTH). VDDR occasionally resembles PHP type 2 in clinical presentation and biochemical features, creating difficulties in the differential diagnosis of these two entities. Here we report an infant diagnosed with VDDR. In addition to inadequate vitamin D intake, usage of antiepileptic drugs (AED) may have led to the worsening of the vitamin D deficiency. The patient presented with a history of febrile convulsions, for which he received phenobarbital treatment. The initial findings of hypocalcemia, hyperphosphatemia and normal tubular reabsorption of phosphate, mimicking PHP 2, responded well to vitamin D and oral Ca treatment with normalization of serum Ca, phosphorus (P), ALP and PTH levels

**Conflict of interest:**None declared.

## INTRODUCTION

Vitamin D deficiency rickets (VDDR) remains to be a common disorder in developing countries. In a recent study, it was shown that after the nationwide vitamin D supplementation campaign in 2005, the prevalence of VDDR declined from 6.09% to 0.099% in children aged between 0−3 years in Turkey (1). There are some studies reporting the resurgence of this disorder in developed countries (2, 3).

Pseudohypoparathyroidism (PHP) is an inherited disorder characterized by an end−organ resistance to parathormone (PTH). Children with PHP present with hypocalcemia and normal or elevated serum phosphorus (P) concentrations despite elevated serum PTH levels. The disorder is subdivided into several distinct entities. PHP type 1 is differentiated from PHP type 2 by reduced urinary cAMP excretion in response to administration of PTH.

Pediatricians in developed countries have limited experience in treating children with VDDR (4). PHP and VDDR can be mistaken for one another because of similarity in their presentation and biochemical features.

Here we report a 23−month−old male patient presenting with recurrent febrile convulsions and diagnosed as antiepileptic drug (AED)−induced VDDR. The initial levels of serum Ca, P, PTH and tubular phosphate reabsorption were suggestive of PHP.

## CASE REPORT

A 23−month−old male was brought to our clinic for evaluation of fever. He was born at term with a birth weight of 2500g to nonconsanguineous parents. He had a convulsion for the first time at age 8 months and, antiepileptic phenobarbital treatment was started. The patient had had febrile convulsions on 7 different occasions since then. He was breastfed only until 6 months of age and received complementary feedings thereafter. The infant was given 400 U/day vitamin D supplementation only in the first two months of life. His neurodevelopment was appropriate for age. The patient had a history of frequent infections. He had been hospitalized due to pyelonephritis at the age of 16 months.

On physical examination, his height was 84 cm (25^th^−50^th^ percentile), weight 12.5 kg (50^th^ percentile), head circumference 48 cm (50^th^ percentile). Mild enlargement of the wrists was noted bilaterally. Interpopliteal distance was 5.5 cm (N:<4.5 cm). Rachitic rosary, craniotabes, caput quadratum, and leg deformities (O−/X−bine) were not present. He had also no signs suggestive of Albright’s hereditary osteodystrophy (AHO), such as short hands, round face, central obesity or dental hypoplasia.

Initial laboratory investigations showed the following levels: glucose 104 mg/dL, Na 134 mmol/L, K 4.5 mmol/L, BUN 8 mg/dL, creatinine 0.4 mg/dL, Ca 6.4 mg/dL (N: 8.8− 10.8), P 6.7 mg/dL (N: 3.8−6.5), Mg 0.9 mmol/L (N:0.6−0.95), alkaline phosphatase (ALP): 436 IU/L (N:145−420), total protein 6.5 g/dL (N:6.1−7.9), albumin 4.4 g/dL (N:3.5−5), PTH 390.7 pg/mL (N:11.1−79). Left hand and wrist X−ray showed no findings of rickets. Upon these results, PHP or VDDR was suspected. Urinary Ca/creatinine ratio was 0.26 (N:<0.6). Tubular reabsorption of phosphate was 0.99 (N:>0.90). Aminoaciduria was absent. EEG was normal. Ca replacement was started in a dose of 50 mg/kg/day. The level of serum 25−hydroxyvitamin D [(25(OH)D] was low−4.5 ug/L. A diagnosis of vitamin D deficiency was made and, oral 300 000 U vitamin D was started. In addition, oral Ca was given for 15 days. Three weeks later, serum Ca level was 9.2 mg/dL, P 6 mg/dL, ALP 354 U/L. The level of PTH had decreased to 37 pg/mL, thus, the diagnosis of PHP was excluded. Vitamin D in a dose of 400 IU/day was prescribed. Regular follow−up, including measurement of serum 25(OH)D levels, was recommended.

## DISCUSSION

VDDR is a disorder biochemically characterized by elevated serum ALP activity, normal or decreased serum Ca and inorganic phosphate concentrations, secondary hyperparathyroidism and decreased serum 25(OH)D levels. Fraser et al (5) described three stages of VDDR on the basis of clinical and laboratory data. Stage 1 is characterized by hypocalcemia, normal P, normal urinary amino acid and phosphate excretion with minimal or no findings of rickets on radiographs. Stage 2 is associated with normal serum Ca and low P concentrations in the presence of phosphaturia and aminoaciduria and mild to moderate rickets findings on X−rays. Stage 3 is similar to stage 2 but associated with hypocalcemia and advanced rickets. In this original study, which is based on the classical teaching of rickets staging, hypocalcemia was attributed to lack of PTH secretion in the first stage of VDDR. The earlier studies also showed normal PTH levels in this stage (6). Later on, it was shown that PTH levels were actually increased in stage 1 of VDDR at a time when the phosphaturia or aminoaciduria were still absent. It was also shown that the regulation and action of PTH were disturbed in vitamin D deficiency, and restored after vitamin D treatment (7). Srivastava (4) was the first to draw attention to the possibility that the elevated PTH levels associated with hypocalcemia and normal P indicate an element of end−organ resistance to PTH, mimicking PHP.

PHP is a genetic disorder associated with increased secretion of PTH and target−tissue unresponsiveness to the biological actions of PTH. In PHP patients, biochemical findings (hypocalcemia and hyperphosphatemia) are consistent with hypoparathyroidism. PHP type 1a is characterized by hormone resistance in addition to a peculiar constellation of developmental and somatic defects that are collectively termed as AHO. The AHO phenotype consists of short stature, round face, obesity, brachydactyly, subcutaneous  ssifications, and rarely dental defects and sensorineural abnormalities. Our patient had none of these findings. Similar to patients with PHP type 2, patients with PHP type 1b present with only PTH resistance in the absence of features of AHO. The mutations of GNAS1, a gene encoding the alpha− subunit of the G stimulatory protein, are responsible for the two main subtypes of PHP, namely types 1a and 1b. Type 1c, the rarest subtype, is characterized by AHO as well as resistance to PTH and other hormones associated with normal Gs α activity. In our patient, the clinical findings and initial laboratory results suggested PHP type 2 or PHP type 1b.

There are several studies reporting on the difficulty in differentiating stage 1 VDDR from PHP (4, 8, 9). Serum PTH levels cannot be relied on to differentiate between these two disorders. A detailed history should be taken, including vitamin D intake, sun exposure and use of drugs that interfere with vitamin D metabolism. In our patient, vitamin D support was inadequate and phenobarbital treatment probably aggravated the deficiency. Evidence of rickets on physical and radiological examination is also important in the differential diagnosis, although clinical signs may be minimal or absent in stage 1 VDDR. On the other hand, PHP may present with bony deformities resembling rickets (10). The hallmark of VDDR is vitamin D depletion (low serum 25 (OH)D), since serum Ca, P and 1,25 (OH)2D levels can be variable (7). Nevertheless, if the differential diagnosis cannot be made properly in marginal cases, showing the normalization of biochemical and physical abnormalities after vitamin D treatment may help in clarifying the diagnosis (4). Although the clinical and radiological findings were vague in our patient, the 25 (OH)D level was found low and stoss therapy (300 000 IU vitamin D, orally) led to normalization of serum Ca, P, ALP and PTH in three weeks.

Febrile seizures, a common and usually benign complaint in infancy and early childhood, are not known to be associated with rickets. Hoecker et al (11) reported that recurrent febrile convulsions might be an unusual presentation of nutritional rickets. Since vitamin D is reported to have an important role in immune defense mechanisms (12), it may be speculated that the febrile convulsions triggered by fever during frequent infections in this patient could be attributable to vitamin D deficiency.

There are several studies reporting that decreased serum 25(OH)D and altered bone metabolism were associated with AED treatment in children (13). Many AEDs are known to be inducers of hepatic cytochrome P450 metabolism, resulting in increased hepatic metabolism of vitamin D. Some authors recommend monitoring of the vitamin D status in children taking AEDs, especially those receiving polypharmacotherapy (14). In our patient, we thought that in addition to inadequate vitamin D intake, usage of AED may have led to the worsening of the vitamin D deficiency.

In conclusion, reporting this case we desire to emphasize that VDDR can be mistaken for PHP in some cases. The demonstration of normalization of phosphocalcic parameters (i.e. serum Ca, P, ALP and PTH) after vitamin D treatment may be useful in the differentiation of these two disorders. Vitamin D deficiency should be kept in mind in the differential diagnosis of hypocalcemia in children, especially those being treated with AEDs.
